# Mechanical Force Regulates the Paracrine Function of ADSCs to Promote the Adipose‐Regenerating Effects of AAM by Regulating Angiogenesis and the Inflammatory Response

**DOI:** 10.1111/cpr.70045

**Published:** 2025-04-27

**Authors:** Yining Wang, Luyu Zhang, Jiaxuan Liu, Yuchao Yang, Zhenyu Bi, Jun Ouyang

**Affiliations:** ^1^ Guangdong Provincial Key Laboratory of Digital Medicine and Biomechanics, Guangdong Engineering Research Center for Translation of Medical 3D Printing Application, National Key Discipline of Human Anatomy, Department of Anatomy, School of Basic Medical Sciences Southern Medical University Guangzhou P. R. China; ^2^ Yue Bei People's Hospital Postdoctoral Innovation Practice Base Southern Medical University Guangzhou P. R. China; ^3^ Department of Gastroenterology The First Affiliated Hospital of Anhui Medical University Hefei P. R. China

## Abstract

Conditioned medium (CM), obtained by mechanical regulation of the paracrine activity of ADSCs, was fused with acellular adipose matrix (AAM) and methyl cellulose (MC) to synthesize a composite hydrogel which was grafted onto nude mice. The composite hydrogel could promote soft tissue regeneration by regulating the level of vascular regeneration and inflammation.

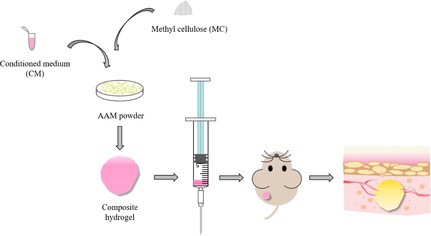

Soft tissue defects are a common complication in pathological trauma and its treatment. Although numerous methods promoting soft tissue regeneration in vivo to treat soft tissue defects have been explored, a gold standard remains elusive owing to various limitations. At present, autologous grafts or synthetic polymers are most frequently used to treat soft tissue defects [1]; however, their effectiveness is constrained by challenges such as donor site injury, transplantation failure, and poor mechanical properties. The emergence of tissue engineering technology has provided new possible solutions.

Tissue engineering scaffolds can provide physical and biological support for new tissue formation as well as offer a suitable microenvironment for tissue regeneration. Acellular adipose matrix (AAM), as a tissue engineering scaffold material with low antigenicity and high biocompatibility, provides a highly conducive milieu for fat formation, both in vitro and in vivo [2]. AAM retains the complex structure and composition of extracellular matrix (ECM), including collagen, elastin, and adhesion peptides, as well as sophisticated substances to promote cell adhesion and proliferation by providing a fat‐like microenvironment for adipose tissue survival. Furthermore, AAM forms new adipose tissue whose morphology is similar to the natural morphology of adipose cells in natural adipose tissue. In clinical settings, AAM is increasingly being used to generate injectable hydrogels, whose use as clinical soft tissue fillers offers a minimally invasive strategy for treating soft tissue defects.

Theoretically, soft tissue substitutions without seeding of exogenous cells could induce in situ adipose regeneration, which is sufficient to repair defects [3, 4, 5]. However, in practical application, the effect of AAM in promoting soft tissue regeneration is still limited; this is mainly because this material lacks suitable mechanical and biochemical properties [6]. In previous studies, in order to improve the mechanical properties of AAM [7, 8, 9, 10], Methyl cellulose (MC) was mixed with AAM to produce an injectable hydrogel, which was proven to promote adipose regeneration. Our research group further explored the regulating effect of MC on the mechanical properties of the AAM scaffold and found that the stiffness of AAM can be adjusted to the most suitable level for adipose regeneration, at a concentration of 0.075 g/mL MC [8]. Therefore, in this study, this concentration was used to prepare the novel hydrogel.

Furthermore, to compensate for the lack of suitable biochemical characteristics of AAM, the integration of growth factors or cytokines into bioactive scaffolds is a useful strategy [3, 11, 12]. The formation of stable soft tissue at the recipient site requires sufficient angiogenesis and adipose tissue regeneration, which is a major challenge [13]. Integrating growth factors or ECM proteins into scaffolds is helpful in promoting angiogenesis and a lipogenic microenvironment. Angiogenesis is of great significance for fat regeneration. During adipose tissue development and maintenance, there is a strong correlation between adipogenesis and angiogenesis. An insufficient vascular supply will lead to insufficient adipose formation [14]. A previous study suggested that active components such as VEGF and bFGF might underlie the ability of the composite hydrogel to exhibit stronger effects on adipogenesis [14, 15].

Adipose‐derived stem cells (ADSCs) can promote soft tissue regeneration through direct differentiation into appropriate cell types and paracrine regulation. ADSCs secrete a variety of cytokines and growth factors, promote angiogenesis, enhance endogenous stem cell recruitment, and regulate the inflammatory response, which may be useful to coordinate in situ tissue regeneration [16]. Previous studies have shown that mechanical stimulation can not only regulate the migration and differentiation of ADSCs, but also modulate their paracrine function and induce the secretion of various growth factors by ADSCs [17, 18, 19]. Therefore, we collected the conditional medium (CM) produced by ADSCs that were exposed to different extents of mechanical stretching deformation, and then detected their angiogenic and inflammatory factors by ELISA. The results indicated that 15% stretch deformation regulates the paracrine secretion of ADSCs, resulting in increased production of angiogenic factors (VEGF, bFGF) and reduced secretion of inflammatory factors (IL‐6, IL‐8), which is more suitable for the synthesis of composite hydrogels (Figure S1). Controlling the inflammatory reaction of biomaterials can prevent the degradation of biomaterials before successful tissue regeneration and avoid deterioration and/or chronic inflammation [20]. Sufficient autologous vascularised tissue can provide nutrition for adipocytes, which is beneficial to maintain adipose volume for a long time [21]. Therefore, we used the above CM to promote the adipose regenerative effects of the mechanical hydrogel.

Besides angiogenesis, inflammation also plays an important role in regulating the metabolic homeostasis of adipose tissue. Increasing evidence shows that adipose tissue macrophages, especially the M2 phenotype, are major immune cell types in adipose tissues that are thought to play important roles in adipose tissue remodeling [22, 23, 24]. A previous study found that in the process of inducing lipogenesis, most of the cells that migrate first are macrophages, while fat precursor cells account for only a few [22]. The recruitment of endogenous macrophages into the hydrogel induces adipose tissue remodeling and adipogenesis [25].

In general, we designed a novel injectable hydrogel that can be used to fill irregular defects and stimulate natural soft tissue regeneration. CM obtained from regulating the paracrine function of ADSCs by mechanical tension was fused with AAM and MC to synthesize a composite hydrogel (Figure 1). On the one hand, MC was used to adjust the stiffness of the hydrogel to provide a mechanical environment consistent with normal adipose tissue; On the other hand, CM could assist AAM to provide a regenerative microenvironment for soft tissue repair. The purpose of this study is to evaluate the potential of this novel injectable composite AAM hydrogel to promote tissue regeneration by regulating the level of vascular regeneration and inflammation.

**FIGURE 1:**
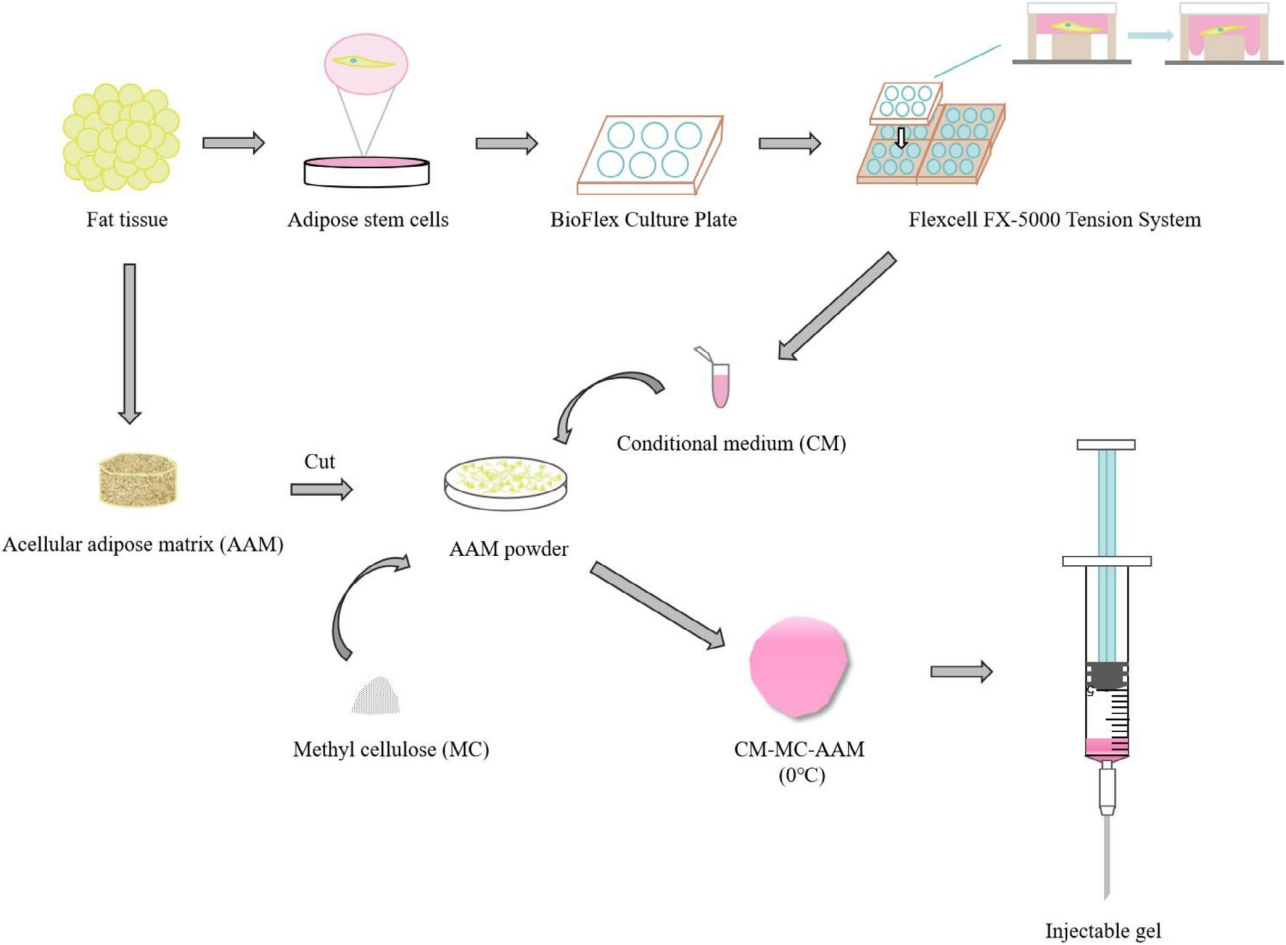
Preparation of injectable composite hydrogel.

In this paper, we first tested the mechanical properties of the composite hydrogel in vitro. The hydrogel composed of CM, AAM, and MC was defined as the CM group. The NC group used DMEM basic medium instead of CM. Observation by the naked eye and electron microscopy revealed no obvious structural differences between the composite hydrogels of the CM group and the NC group(Figure 2a). Under a transmission electron microscope, the two groups of composite hydrogels were observed to have dense fiber networks (Figure 2b). Comparison of the mechanical properties of the two groups of composite hydrogels revealed no significant differences in stiffness and rheological properties between the CM group and the NC group (Figure 2c,d).

**FIGURE 2:**
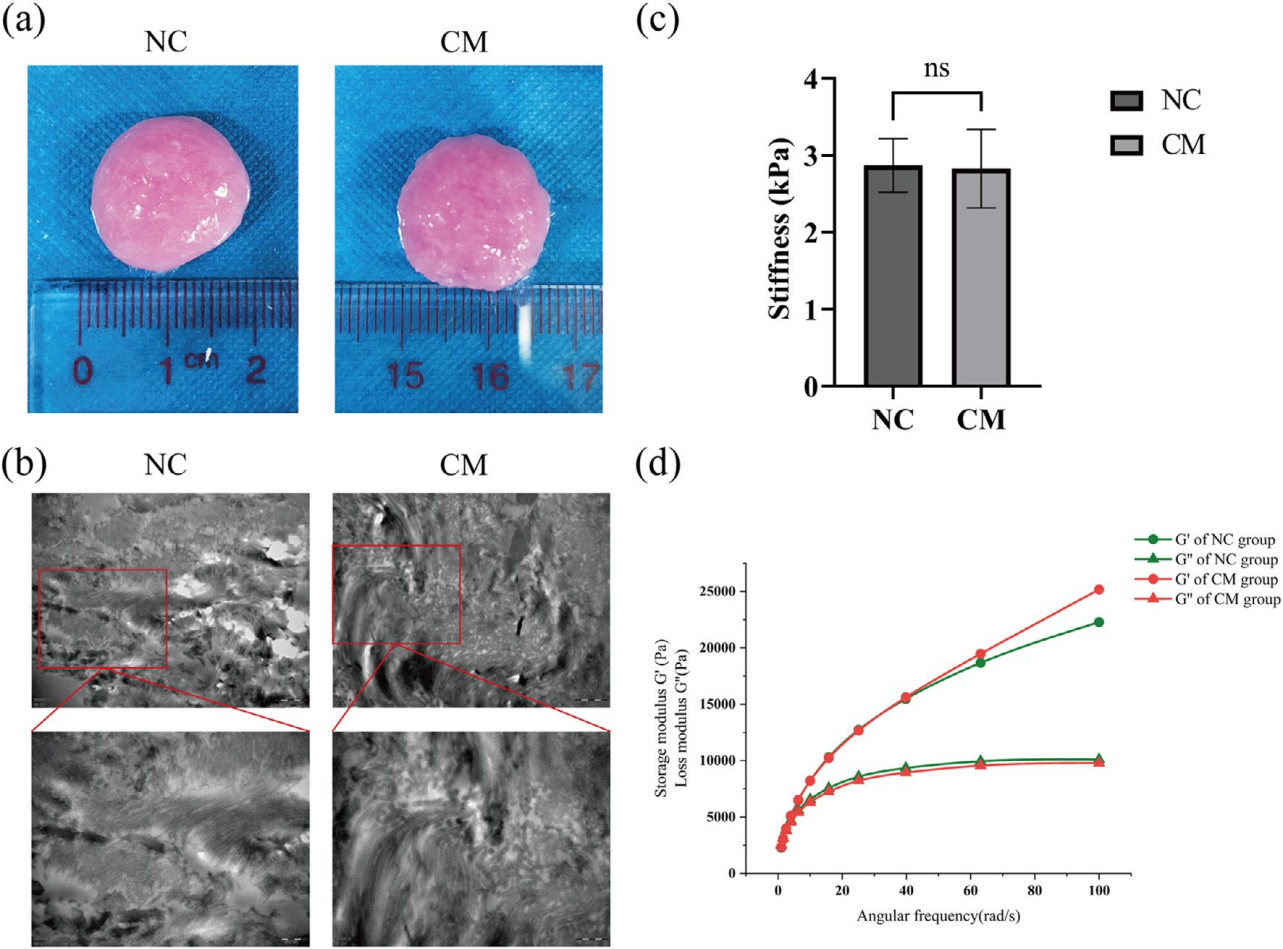
Gross morphologies, stiffnesses, and microstructures of composite hydrogels. (a) Top views of the NC group and CM group of composite hydrogels. (b) Transmission electron microscope images of the two groups of composite hydrogels. The red boxes are magnified in the lower images. Scale bar: 1 μm, 500 nm. (c)Stiffness analysis of the two group of composite hydrogels (ns, no significance). (d) Storage modulus and loss modulus of two groups of composite hydrogels.

Two groups of composite hydrogels were transplanted into the backs of nude mice, and the grafts were extracted for testing on days 3, 7, 14, and 30 (Figure 3a). Each implant was characterized by a fibrotic capsule around the compound (Figure 3b). Overall, the graft retention rate in the CM group was higher than that in the NC group. The retention rate of the graft decreased with time, and on the 30th day, the texture of the graft had changed from hard to extremely soft (Figure 3c). The results of immunohistochemistry (IHC) showed that the novel composite hydrogel could promote early capillary regeneration, which is beneficial to adipogenesis. It showed that in the early stage of transplantation, the CD31‐positive area in the CM group was quantifiably higher than that in the NC group, especially on the 7th day (*p* < 0.0001). On the 30th day, the CD31‐positive area in the NC group was quantifiably higher than in the CM group; however, the difference was not significant (Figure 3d,e).

**FIGURE 3:**
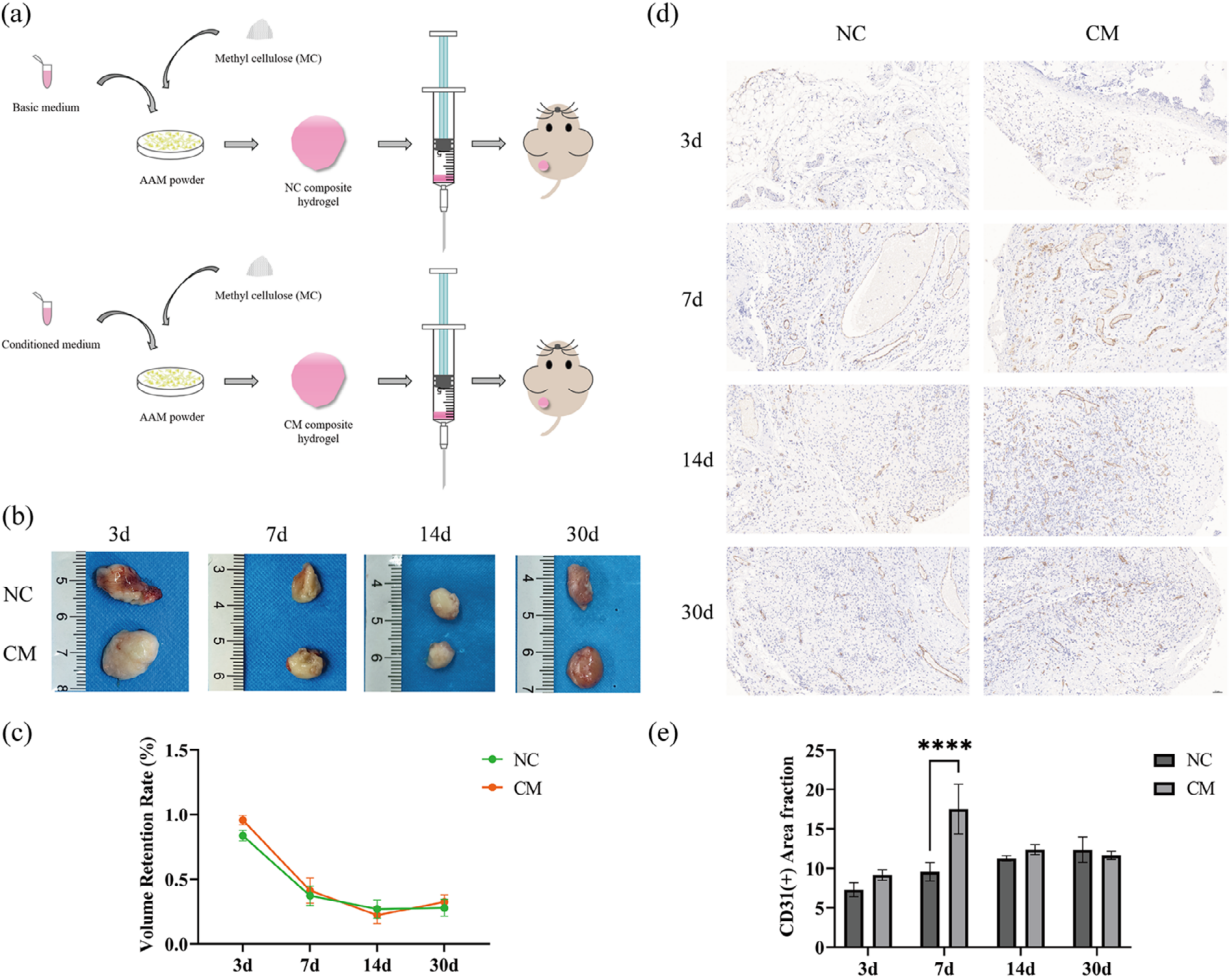
Sample volume and vascularization of grafts. (a) Schematic illustration of the setup of the animal experiment. (b) Gross morphology of grafts in the two groups on days 3, 7, 14, and 30 after subcutaneous injection into nude mice. (c) Percentage volume retention of the grafts in each group on days 3, 7, 14, and 30. (d) Immunohistochemistry staining of CD31 (brown) in the two groups on days 3, 7, 14, and 30. (e) Quantification of CD31‐positive areas (number of capillaries per optical field) in grafts in the two groups on days 3, 7, 14, and 30 (****p < 0.0001). Scale bar: 50 μm.

Hematoxylin–eosin and Masson's trichome staining of the grafts on days 3, 7, 14, and 30 after injection into nude mice showed that the grafts were denser and collagen had formed earlier in the CM group, indicating a higher level of fibrosis in the graft. The cell density of the graft in the CM group was higher on the 14th day, while that in the NC group was higher on the 30th day (Figure 4).

**FIGURE 4:**
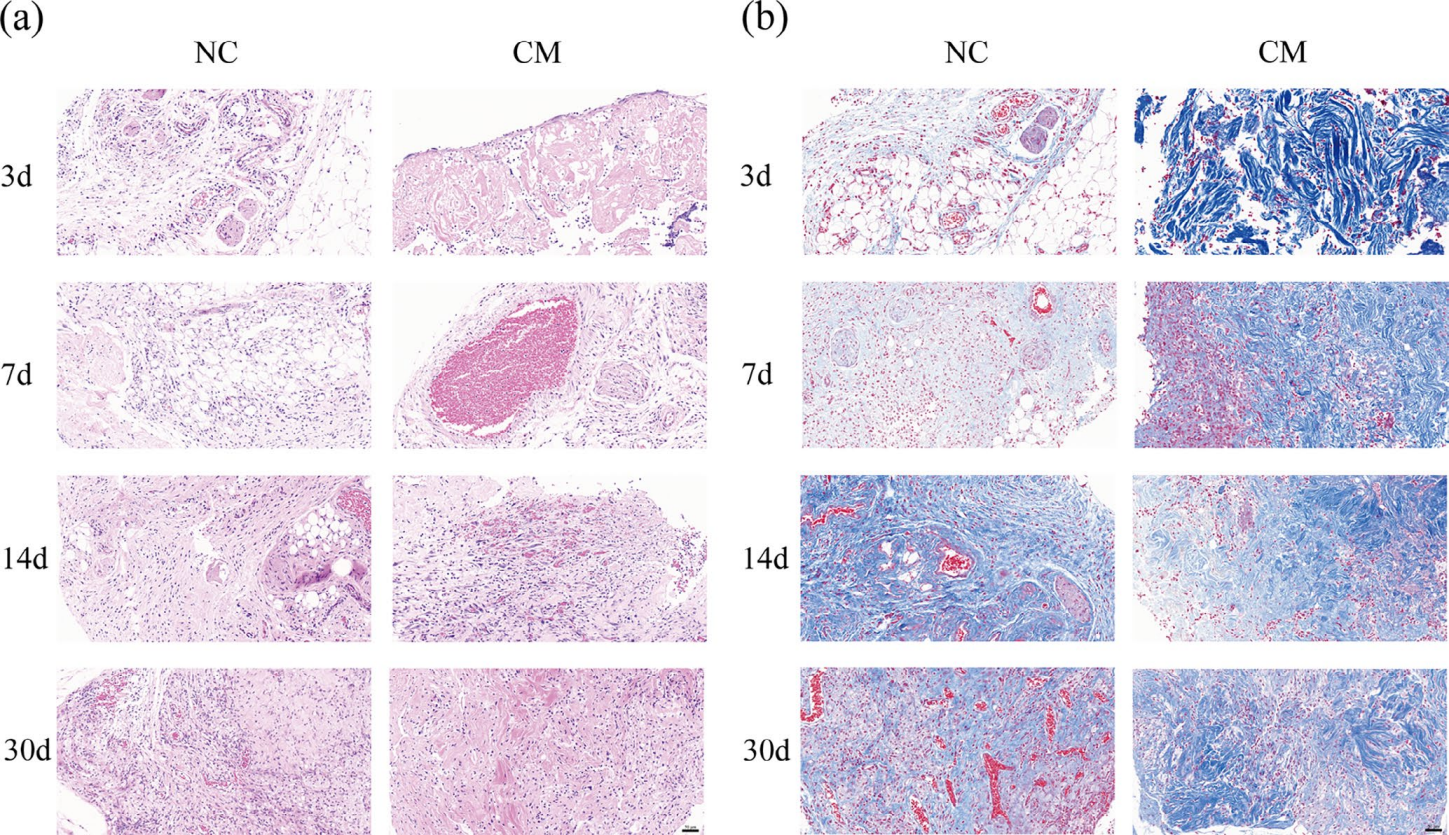
Histological analysis of grafts in the two groups on days 3, 7, 14, and 30. (a) Hematoxylin–eosin staining of grafts. (b) Masson's trichome staining of grafts. Scale bar: 50 μm.

The novel composite hydrogel effectively promotes adipose regeneration. The CM group appeared to contain more perilipin‐positive areas than the NC group on days 14 and 30. Quantitative analysis of the stained samples confirmed these visual observations (all *p* < 0.05) (Figure 5a,b). Enhancing the activity of enhancer binding protein β (C/EBP β) and peroxisome proliferator activated receptor‐γ (PPAR‐γ) could induce peripheral preadipocytes [6]. The results of WB analysis showed that the novel composite hydrogel could effectively improve the expression of PPAR‐γ, adipocyte protein‐2 (aP2 α), and C/EBP β (Figure 5c). Compared with that in the NC group, the expression of PPAR‐γ1 and PPAR‐γ2 in the CM group was significantly higher (all *p* < 0.05) on day 30, and higher—but not significantly so—on day 14 (Figure 5d,e). The expression of aP2 α in the CM group was higher than that in the NC group on day 14 and day 30 (*p* < 0.01, *p* < 0.001) (Figure 5f). Compared with that in the NC group, the expression of C/EBP β in the CM group was significantly higher on day 30 (*p* < 0.05) and higher—but not significantly so—on day 14 (Figure 5g).

**FIGURE 5:**
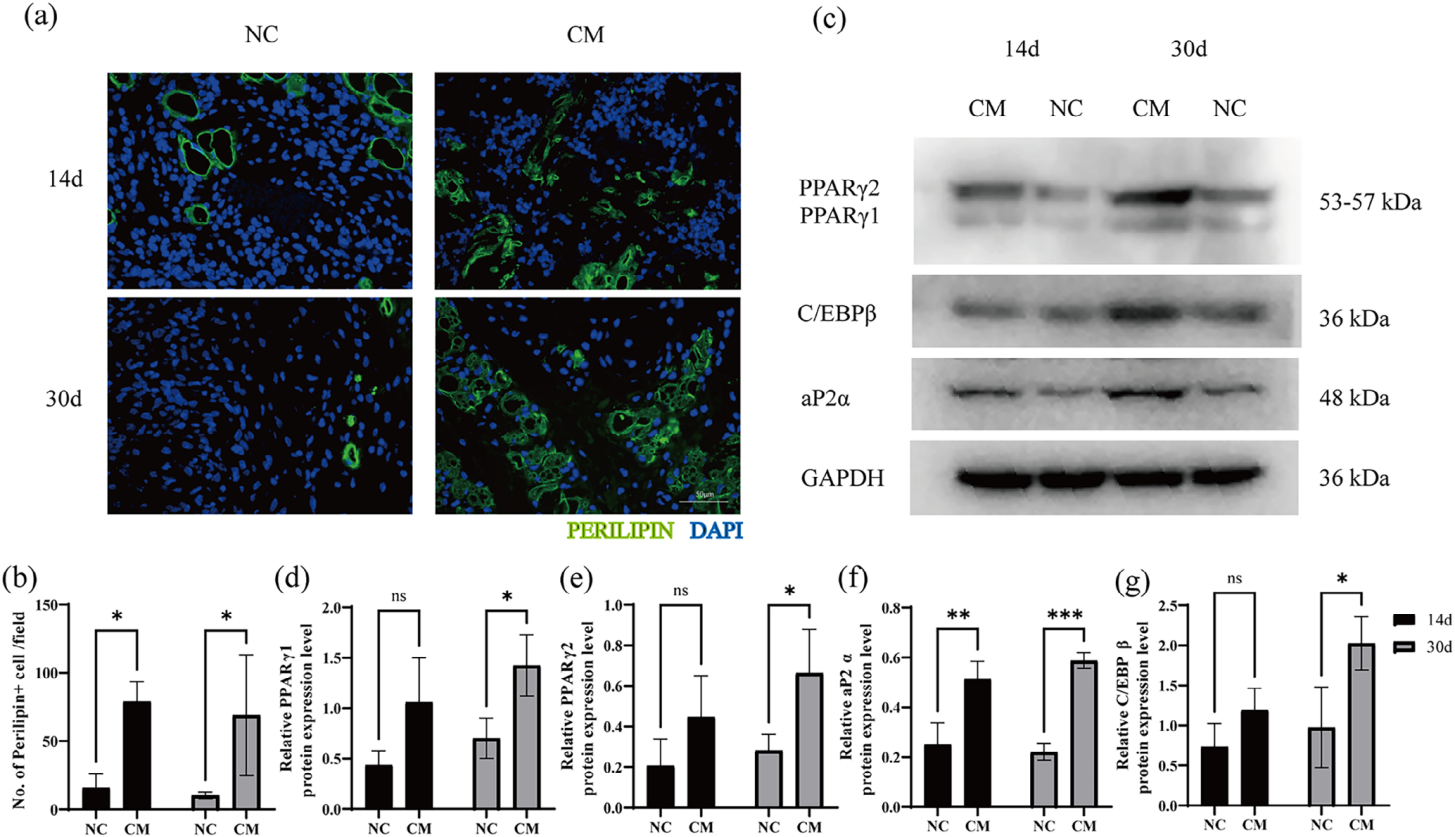
Adipogenesis and expression of adipogenesis‐associated proteins. (a) Immunofluorescence staining of perilipin (green) and DAPI (blue) in the two groups on days 14 and 30. Scale bar = 50 μm. (b) Quantification of perilipin‐positive cells in grafts from the two groups on days 14, and 30. (c) Western blot analyzes of PPAR γ, aP2 α, and C/EBP β in grafts from the two groups on days 14 and 30. (d‐g) Quantification of the PPAR γ, aP2 α, and C/EBP β band intensities, respectively, in the two groups on days 14 and 30. (*p < 0.05, **p < 0.01, ***p < 0.001; n.s, no significance).

In this study, the novel composite hydrogel was found to promote adipose regeneration by regulating the inflammatory reaction. Immunofluorescence staining of inflammatory cells demonstrated that the proportion of MAC2(+)&CD206(−) M1 macrophages in the CM group was significantly lower than that in the NC group on day 7 (*p* < 0.001) and day 14 (*p* < 0.05). This may indicate that the graft reduced the number of M1 macrophages, which means the polarization of macrophages is shifted to the M2 phenotype, to attenuate the inflammatory reaction (Figure 6a,b). Results from the RT‐qPCR showed that the relative expression levels of TNF‐α (*p* < 0.0001), IL‐6 (*p* < 0.001), and IL‐8 (*p* < 0.0001) in the CM group were significantly higher than those in the NC group on day 3. On the 7th day, the relative expression levels of inflammatory factors in the two groups increased, and the relative expression levels of TNF‐α (*p* < 0.0001), IL‐6 (*p* < 0.0001), and IL‐8 (*p* < 0.0001) in the CM group were still higher than those in the NC group. However, with the passage of time, the relative expression level of inflammatory factors in the CM group was significantly lower, which may be related to the obvious decrease in the proportion of M1 macrophages in the CM group. On day 14, there was no significant difference in the relative expression levels of TNF‐α between the CM group and the NC group. In addition, the relative expression level of IL‐6 (*p* < 0.01) was significantly lower than that in the NC group, while the relative expression level of IL‐8 (*p* < 0.01)was slightly higher than that in the NC group. Until the 30th day, there was no significant difference in TNF‐α, IL‐6, and IL‐8 between the two groups (Figure 6c–e). Although the CM we used to prepare the novel composite hydrogel contained IL‐6 and IL‐8 (Figure S1), after transplantation, the composite hydrogel inhibited the inflammatory reaction through the high expression of M2 macrophages, which contributed to adipose remodeling and vasculogenic regeneration.

**FIGURE 6:**
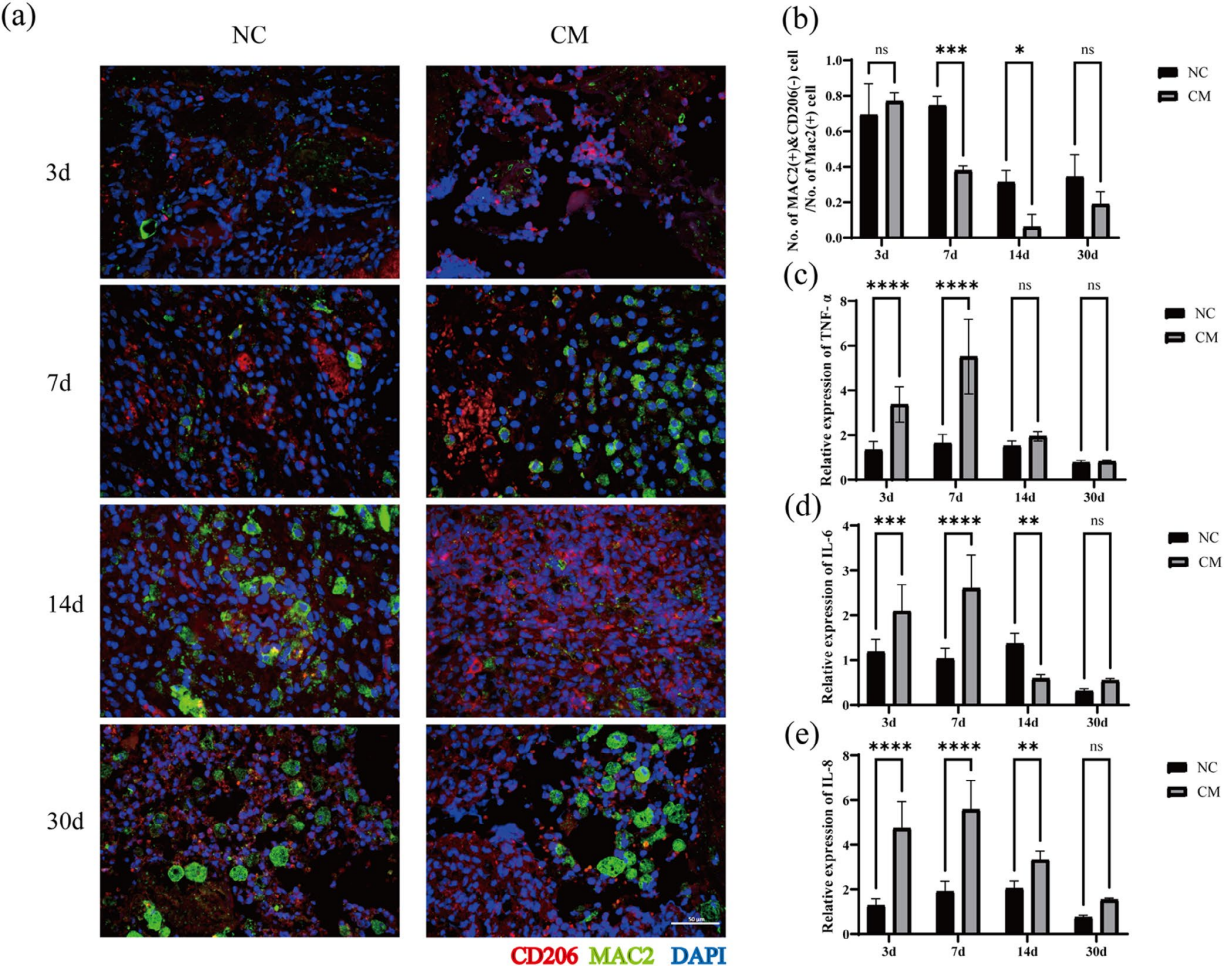
Macrophage infiltration and relative expression of inflammation‐related factors. (a) Immunofluorescence staining of CD206 (red), MAC2 (green), and DAPI (blue) in the two groups on days 3, 7, 14 and 30. Scale bar = 50 μm. (b) The proportion of MAC2 (+)&CD206 (−) M1 macrophages in all MAC2 (+) macrophages on days 3, 7, 14, and 30. (c–e) RT‐qPCR was carried out to evaluate the relative expression levels of TNF‐α, IL‐6, and IL‐8. (*p < 0.05, **p < 0.01, ***p < 0.001, ****p < 0.0001; n.s, no significance).

In conclusion, we found that the novel composite hydrogel showed bioactive effects on macrophage infiltration and polarization, angiogenic response, and adipogenesis. In particular, the novel hydrogel exhibited apparent pro‐adipogenic and macrophage polarization abilities. However, according to a previous study, the growth factor in CM added to hydrogel is dose‐sensitive to adipose formation [6]. Therefore, it is very important to find an optimal concentration instead of using a large dose. This study initially confirmed that 15% mechanical stretch deformation is the most effective mechanical condition to stimulate ADSCs for adipose formation. In future, more experiments in vivo will be performed to demonstrate the advantages of the novel composite hydrogel for angiogenesis and adipogenesis.

## Author Contributions

**Yining Wang:** conceptualisation, methodology, investigation, writing – original draft, funding acquisition. **Luyu Zhang and Jiaxuan Liu:** investigation, formal analysis. **Yuchao Yang:** methodology, validation, writing – review and editing, funding acquisition. **Zhenyu Bi and Jun Ouyang:** project administration, resources, writing – review and editing. All the authors read and approved the final manuscript in writing.

## Conflicts of Interest

The authors declare no conflicts of interest.

## Supporting information


**Data S1.** Supporting Information.

**Figure S1.** ELISA detection of expression of inflammatory factors IL‐6, IL‐8, bFGF, and VEGF.

## Data Availability

The data that support the findings of this study are available from the corresponding author upon reasonable request.
