# Supplementary material for: Mechanical Force Regulates the Paracrine Function of ADSCs to Promote the Adipose‐Regenerating Effects of AAM by Regulating Angiogenesis and the Inflammatory Response
Source: Cell Prolif. 2025 Apr 27;58(6):e70045. doi: 10.1111/cpr.70045 (PMC12179550; doi:10.1111/cpr.70045)
Supplement: Supplementary file 1 — Data S1. Supporting Information. [file CPR-58-e70045-s001.docx]

**Supplementary Material**

**Materials and methods**

1. Adipose tissue procurement and processing

All experiments were performed according to the principles of the Declaration of Helsinki. Within 2 h, the adipose tissues were processed for isolation of ADSCs or decellularization, using published methods [1]. The preparation process for AAM was in accordance with the previous published articles [2], involving steps such as mechanical disruption, polar solvent extraction, enzymatic digestion, and sterilization.

Cryo-milling of decellularized adipose tissue was performed as follows: Following decellularization, the AAM was snap-frozen in liquid nitrogen, lyophilized, and finely minced with sharp scissors. The minced AAM was then cryomilled using a laboratory ball mill (Sartorius Stedim Mikro-Dismembrator, Goettingen, Germany) at 2500 rpm for 2 min, and stored at 4℃ until further use. Immediately prior to composite hydrogel fabrication, the AAM was sterilized by exposure to UV light for 60 min.

1. Synthesis of injectable composite hydrogel

2.1 Cell stretching and conditioned medium collection

The technical specifications of ADSCs used in this study refer to the published stem cell standards of human mesenchymal stem cells [3]. ADSCs were seeded in a silicone rubber membrane coated with type I collagen (Col I) at a density of 1×10^4^ cells/cm^2^ in complete medium. After continuous culture for 24 h, the medium was removed and 2 mL of fresh DMEM basic medium was added to starve cells. After cell starvation, all samples were exposed to static stretch for 6 h using a Flexcell® FX-5000T™ Tension system (Flexcell International Corporation, Burlington, NC, USA), and the CM was collected. In accordance with previous studies, we selected 10%, 15%, and 20% deformation to stretch cells in order to induce changes in paracellular secretion [4-8].

For the removal of cellular debris, we centrifuged the supernatant at 16,000 g at 4°C for 20 min. Then, a 0.22-μm filter (Merck-Millipore, Darmstadt, Germany) was used to filter the supernatant.

2.2 Mechanical regulation of matrix hydrogel derived from ADSCs

The composite hydrogel with a final volume of 1 ml comprised the following components: AAM, accounting for 50% of the total volume, and MC, with a content of 0.075 g/ml, are quantitatively fused into 1 ml volume of hydrogel by CM. In our previous research [2], these proportions of MC were confirmed to enable the stiffness of AAM to be adjusted for optimal fat regeneration. The novel composite hydrogel was sterilized by ultraviolet irradiation prior to use in subsequent animal experiments.

3. Characterization of composite hydrogel

The surface morphology of the composite hydrogel was observed under a transmission electron microscope (TEM, H-7500, Hitachi). Both storage (G′) and loss modulus (G″) of the hydrogels were determined at 4°C under 1% shear strain with an Anton Paar Rheometer (MCR102; Anton Paar).

A Bose ElectroForce® load-testing device (TA Instruments, New Castle, DE, USA) was used to measure stiffness, as described in our previous study [9]. Specifically, the load cell used to measure forces ranged from 0 to 225 N. The device’s electromagnetic actuator permitted axial travel of 6 mm. Therefore, a preload was applied to remove slack from tissues and induce elongation. Each tissue sample was clamped; then, a preload (25–30 N) was applied followed by ramp loading. The preload was selected based on preliminary testing. The ramp loading rate was set to 8 mm/s and samples were expected to rupture during the application of this loading. The highest force achieved before the sutures tore was electronically recorded for each sample. Finally, the device automatically calculated the sample stiffness.

1. *In vivo* characterization of the composite hydrogel

All studies were carried out in accordance with the China Institutional Animal Care and Use Committee (IACUC) guidelines for the care and use of laboratory animals, and were approved by the Animal Ethics Committee of Southern Medical university (Approval Number: SMUL202410029).

BALB/c-nu mice (4–6 weeks old, male) were randomly divided into 2 groups (3 mice per group), namely, the NC group and CM group. To evaluate the host response to the hydrogels *in vivo*, mice were anesthetized with pentobarbital sodium; then, 200 μL hydrogel was injected into the subcutaneous tissue on the backs of the mice. At 3 days, 7 days, 21 days, and 30 days, the mice were sacrificed and implants were retained for subsequent evaluation of angiogenesis, inflammation, and adipose tissue formation. The implanted hydrogel was sectioned into two parts—one was fixed in 4% paraformaldehyde for H&E staining, Masson’s trichrome staining, immunohistochemistry, and immunofluorescence; while the other part was used for RT-qPCR and WB analysis.

4.1 *In vivo* hydrogel degradation and histocompatibility

At a preset time point, the embedded hydrogels were excised and their volume was measured. The degradation rate = (M0−Mt)/M0 × 100 %. The tissues fixed in 4 % paraformaldehyde were stained with H&E.

4.2 Immunofluorescence (IF) and immunohistochemistry (IHC) assays

Subcutaneously implanted hydrogel samples were fixed in 4 % paraformaldehyde, and the paraffin-embedded tissues were cut into 5-μm thick slices. Stained tissue slices were histologically assessed with Masson’s trichrome and IF staining for CD31 (Servicebio GB11063-2, Wuhan, China) to verify histopathological alterations during angiogenesis. Immunofluorescence staining of CD206 (Servicebio GB125273, Wuhan, China) and MAC2 (Servicebio GB15246, Wuhan, China) was performed to observe the differences between M1 and M2 macrophages in each group. Immunofluorescence staining of perilipin (CST #9349, Danvers, MA, USA) was used to observe the formation of lipid droplets. Sections were then examined under a fluorescence microscope (Nikon, Tokyo, Japan) and photographed with a digital camera, and positive staining was quantified using Image J software.

4.3 Western blot analysis

Subcutaneously transplanted samples were subjected to lysis with RIPA buffer. Protein concentrations were determined with a BCA Kit. Protein was isolated by 10% SDS polyacrylamide gel electrophoresis and transferred onto polyvinylidene difluoride (PVDF) membranes (Millipore, MMAS, USA). After electrophoresis, the PVDF membranes were removed and sealed with 5% (w/v) skimmed milk powder for 1 h. The membrane was incubated with primary antibodies against PPAR γ, C/EBP β, aP2 α, and GAPDH at 4 °C overnight. After washing with TBST (3 times, for 10 min each), the membrane was subjected to incubation for 1 h with secondary antibody at room temperature. Enhanced chemiluminescence (ECL) chromogenic substrate enhanced immunoreactive protein bands, and images of bands were acquired. Data were analyzed in ImageJ.

4.4 Real-time quantitative polymerase chain reaction (RT-qPCR)

RNA was extracted using TRIzol reagent. Synthesized cDNAs were amplified with the EasyScript First-Strand cDNA Synthesis SuperMix. Using cDNA as the template, mRNA expression of *Tnf-α*, *Il-6*, *Il-8*, and *Gapdh* were detected by quantitative real-time PCR (qRT-PCR). We used an ABI PRISM® 7500 Sequence Detection System to perform qRT-PCR. *Gapdh* was used as the internal reference. The gene expression of specific target genes was quantified using the 2^−ΔΔCt^ method. The primers for target genes are as follows.

*Tnf-α*: F, 5′ -CGATGGGTTGTACCTTGTCT and R, 5′-GGCAGAGAGGAGGTTGACTT;

*Il-6*: F, 5′-GTTGCCTTCTTGGGACTGAT and R, 5′-TTTCCACGATTTCCCAGAGA;

*Il-8*: F, 5′- GGACGATAATCAGGACACCG and R, 5′-AAGCCTACACACAGTCCTCT;

*Gapdh*: F, 5′- GGCCTCCAAGGAGTAAGAAA and R, 5′-GCCCCTCCTGTTATTATGG.

5. Statistical analysis

All numerical data are expressed as the mean±standard deviation (SD). Statistical analyses were performed using GraphPad Prism (GraphPad Software, San Diego, CA), by two-way ANOVA. Values of *p* < 0.05 were considered to indicate statistical significance.

**References**

1. Flynn LE. The use of decellularized adipose tissue to provide an inductive microenvironment for the adipogenic differentiation of human adipose-derived stem cells. *Biomaterials*. 2010; 31(17): 4715-4724.
2. Li Y, Bi X, Wu M, et al. Adjusting the stiffness of a cell-free hydrogel system based on tissue-specific extracellular matrix to optimize adipose tissue regeneration. *Burns Trauma*. 2023; 11: tkad002.
3. Chen X, Huang J, Wu J, et al. Human mesenchymal stem cells. *Cell Prolif*. 2022; 55(4):e13141.

[4] Xue Z, Hu D, Tang H, et al. Mechanical force regulates the paracrine functions of ADSCs to assist skin expansion in rats. *Stem Cell Res Ther*. 2024; 15(1):250.

[5] Lin L-Q, Zeng H-K, Luo Y-L, et al. Mechanical stretch promotes apoptosis and impedes ciliogenesis of primary human airway basal stem cells. *Respir Res*. 2023; 24(1):237.

[6] Shan S, Fang B, Zhang Y, et al. Mechanical stretch promotes tumoricidal M1 polarization *via* the FAK/NF‐κB signaling pathway. *FASEB J*. 2019; 33(12):13254-13266.

[7] Liu J, Li Q, Liu S, et al. Periodontal ligament stem cells in the periodontitis microenvironment are sensitive to static mechanical strain. *Stem Cells Int*. 2017; 2017:1380851.

[8] Ma H, Wang L, Sun H, et al. MIR-107/HMGB1/FGF-2 axis responds to excessive mechanical stretch to promote rapid repair of vascular endothelial cells. *Arch Biochem Biophys*. 2023; 744:109686.

[9] Li Y, Wu M, Zhang Z, et al. Application of external force regulates the migration and differentiation of adipose-derived stem/progenitor cells by altering tissue stiffness. *Tissue Eng Part A*. 2019; 25(23-24):1614-1622.
