# Supplementary figures and images for: Mechanical Force Regulates the Paracrine Function of ADSCs to Promote the Adipose‐Regenerating Effects of AAM by Regulating Angiogenesis and the Inflammatory Response
Source: Cell Prolif. 2025 Apr 27;58(6):e70045. doi: 10.1111/cpr.70045 (PMC12179550; doi:10.1111/cpr.70045)

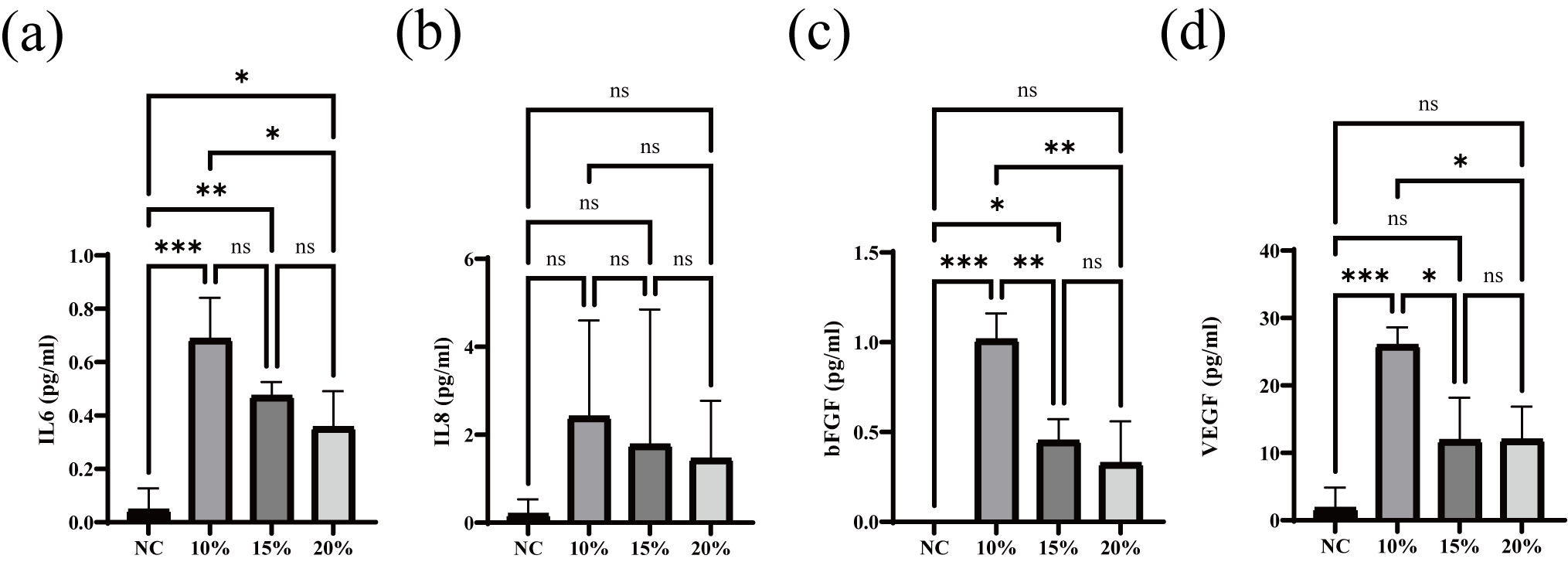


Figure S1. ELISA detection of expression of inflammatory factors IL-6, IL-8, bFGF, and VEGF.

Supplement: Supplementary file 2 — Figure S1. ELISA detection of expression of inflammatory factors IL‐6, IL‐8, bFGF, and VEGF. [file CPR-58-e70045-s002.docx]
